# Regulation of diurnal energy balance by mitokines

**DOI:** 10.1007/s00018-020-03748-9

**Published:** 2021-01-19

**Authors:** Susanne Klaus, Carla Igual Gil, Mario Ost

**Affiliations:** 1 Department of Physiology of Energy Metabolism, German Institute of Human Nutrition Potsdam-Rehbrücke, Nuthetal, Germany; 2 University of Potsdam, Institute of Nutritional Science, Potsdam, Germany; 3 Department of Neuropathology, University Hospital Leipzig, University Leipzig, Leipzig, Germany

**Keywords:** Mitochondria, FGF21, GDF15, Circadian rhythm, Hormones, Nutrition

## Abstract

The mammalian system of energy balance regulation is intrinsically rhythmic with diurnal oscillations of behavioral and metabolic traits according to the 24 h day/night cycle, driven by cellular circadian clocks and synchronized by environmental or internal cues such as metabolites and hormones associated with feeding rhythms. Mitochondria are crucial organelles for cellular energy generation and their biology is largely under the control of the circadian system. Whether mitochondrial status might also feed-back on the circadian system, possibly via mitokines that are induced by mitochondrial stress as endocrine-acting molecules, remains poorly understood. Here, we describe our current understanding of the diurnal regulation of systemic energy balance, with focus on fibroblast growth factor 21 (FGF21) and growth differentiation factor 15 (GDF15), two well-known endocrine-acting metabolic mediators. FGF21 shows a diurnal oscillation and directly affects the output of the brain master clock. Moreover, recent data demonstrated that mitochondrial stress-induced GDF15 promotes a day-time restricted anorexia and systemic metabolic remodeling as shown in UCP1-transgenic mice, where both FGF21 and GDF15 are induced as myomitokines. In this mouse model of slightly uncoupled skeletal muscle mitochondria GDF15 proved responsible for an increased metabolic flexibility and a number of beneficial metabolic adaptations. However, the molecular mechanisms underlying energy balance regulation by mitokines are just starting to emerge, and more data on diurnal patterns in mouse and man are required. This will open new perspectives into the diurnal nature of mitokines and action both in health and disease.

## Introduction

Central to the survival of all living organism is their ability to maintain a balanced energy homeostasis. Organisms are not only able to adjust their energy intake to their metabolic energy requirements, most of them are also able to adapt their metabolic rate to the available energy input, at least within certain limits. Because of their high basal metabolic rate, mammalian metabolism relies on a constant cellular energy influx. Cellular energy demands show a high degree of variability according to cell type and functionality. Muscle energy expenditure for example can increase over 1000-fold during intense exercise, necessitating an integrated response from several organ systems to meet the energy demands of muscle cells [1]. Food (energy) intake occurs in distinct meals and is thus episodic, whereas energy expenditure is both continuous and highly variable. Therefore, mammals including humans have evolved a highly efficient system of metabolic regulation to coordinate energy storage and conversion systems.

Living organisms are subjected to the earth’s 24 h day/night cycle and most mammals occupy distinct temporal niches with the most obvious distinction between day-active species such as humans and night-active (nocturnal) species such as most rodents including mice and rats, the main laboratory model mammals [2]. Accordingly, behavioral as well as metabolic traits show distinct diurnal (24 h) oscillations in most animal species. On a cellular level, nearly all cells of prokaryotic and eukaryotic organisms display circadian rhythms driven by circadian clocks as internal, predictive time-keeping systems [3]. The term circadian defines a self-sustained rhythmicity of approximately (“circa”) 24 h, i.e., a day length (“dia”) which can be synchronized by environmental cues called “zeitgebers” such as the light–dark cycle or temperature rhythms, but also by food intake rhythms [4, 5]. The daily rhythmicity of metabolic processes is driven by the central clock in the suprachiasmatic nucleus (SCN) in the brain, which synchronizes the so-called peripheral clocks existing in most tissues. In turn, metabolic traits also feed back on the circadian system, thus ensuring the flexibility necessary for metabolic and systemic adaptations in response to environmental challenges.

Food, i.e., energy intake, is essential for survival of all animals and it is regulated by both homeostatic and circadian mechanisms which interact continuously. The daily eating pattern is controlled by circadian clocks which interact with feeding related orexigenic and anorexigenic cues including absorbed nutrients, metabolic hormones, and visceral outputs [5, 6]. The metabolic consequences of rhythmic feeding-fasting cycles thus play a dominant role as synchronizing signals for peripheral clocks and it is well recognized that circadian misalignments of the feeding-fasting cycle, e.g., by shift work, can lead to metabolic disorders such as obesity and associated pathologies. There are numerous and excellent recent reviews covering the intricate relationship between the circadian system and energy metabolism [6–9], and here we will focus on the less well covered role of mitochondria in the diurnal regulation of energy balance, especially the emerging role of mitokines as mediators of mitohormesis.

Mitochondria are crucial organelles for cellular energy generation and biosynthetic pathways and also for intra- and inter-cellular signaling. The circadian nature of mitochondrial biology and its control by the circadian clock system is well recognized. This applies to various aspects of mitochondrial biology such as biogenesis, molecular composition, morphological dynamics, respiration, and redox homeostasis [10, 11]. Newly emerging evidence suggests that mitochondrial status might also feed back on the circadian system possibly via mitokines that have been shown to play a role in the diurnal regulation of systemic energy balance. Mitokines are signaling factors that are induced by mitochondrial stress and are considered as endocrine-acting agents of mitohormesis, the induction of cytoprotective pathways leading to an increased stress resistance [12]. Mitohormesis pathways are thought to preserve not only cellular function and survival but also to affect and improve systemic energy metabolism with health promoting consequences [13, 14]. Fibroblast growth factor 21 (FGF21) and growth differentiation factor 15 (GDF15) are two endocrine-acting mitokines with well-established metabolic actions and distinct effects on overall energy metabolism. Both are induced as mitokines in different mouse models of skeletal muscle dysfunction such as the Deletor mouse, a model for mitochondrial myopathy [15] and UCP1-transgenic (tg) mice displaying metabolic adaptations and altered daily fluctuations of substrate metabolism driven by slight skeletal muscle mitochondrial uncoupling [12, 16, 17]. Here we will review the role of these mitokines in the diurnal regulation of energy homeostasis with a special emphasis on GDF15 and its recently uncovered role as a novel player in the diurnal regulation of feeding patterns and energy balance.

## The importance of energy balance regulation

**Fig. 1:**
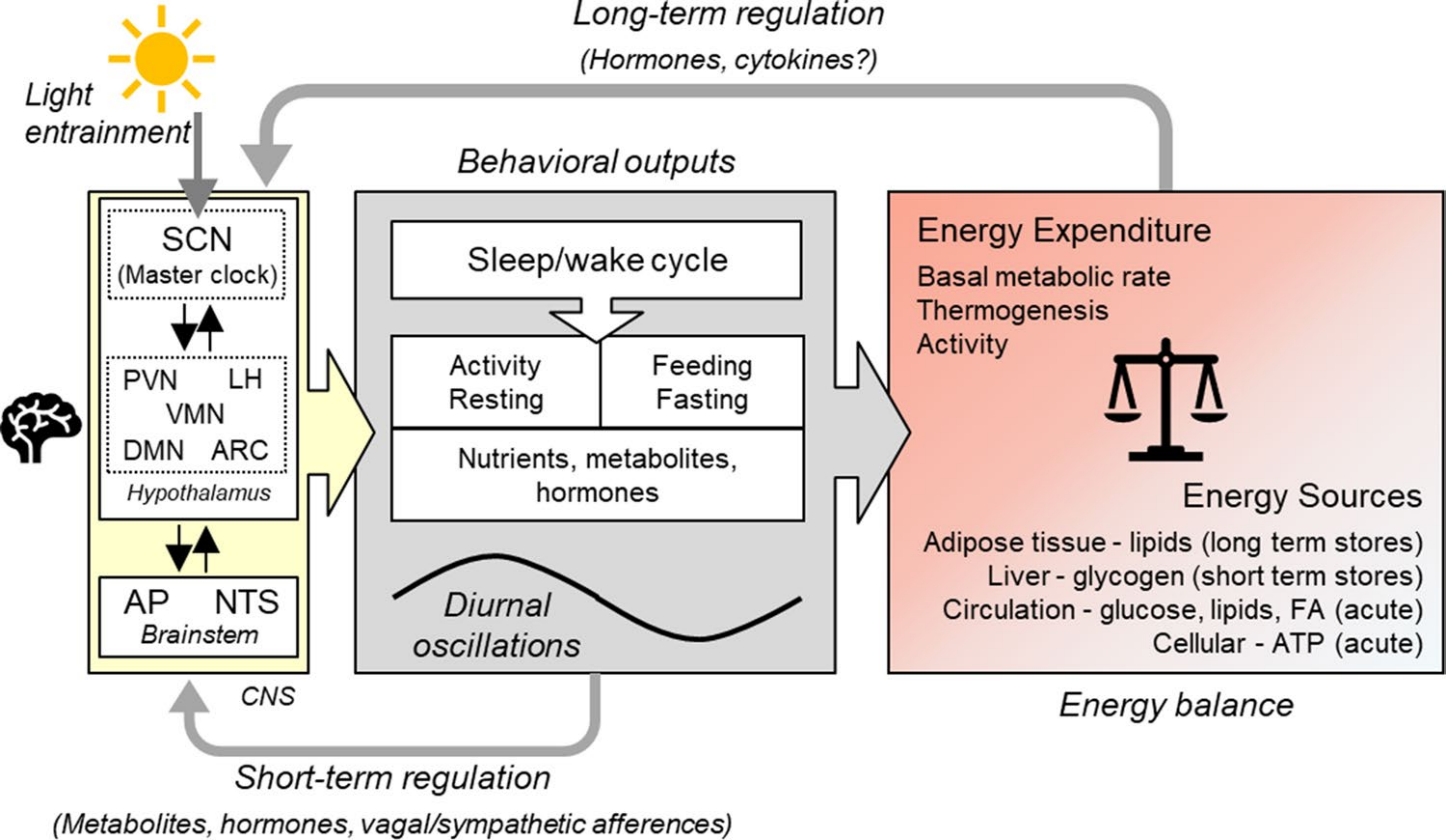
Systemic regulation of energy homeostasis. Systemic energy balance is driven by an equilibrium between energy expenditure, i.e., energy consuming metabolic processes and energy supply, which necessitates the availability of acute, short, and long-term energy sources. Replenishment of energy stores occurs by feeding, and both, feeding/fasting and activity/resting, are behaviors that show diurnal oscillations leading to diurnal fluctuations in circulating nutrients, metabolites and hormones. These diurnal behaviors are mainly driven by the sleep wake cycle governed by the circadian rhythmicity of the hypothalamic SCN master clock. This central pacemaker, which is entrained by light via retinal input and thus synchronized to the natural light dark cycle, coordinates peripheral rhythms through neuronal and humoral outputs. The SCN is bidirectionally connected to other hypothalamic nuclei that are involved in appetite regulation and energy homeostasis. Functional connections also exist between the hypothalamus and the brainstem, another important brain center for energy homeostasis and appetite regulation, that receives direct input from the periphery via hormonal and nervous signals. Short term regulation refers to episodic signals relating to food intake and satiation such as intestinal vagal afferences and gut hormones (gut-brain axis), whereas long term regulation refers to hormonal signals conveying information about body energy stores most prominently exemplified by the adipokine leptin, which signals the amount of body fat stores. *AP* area postrema, *ARC* arcuate nucleus, *DMN* dorsomedial nucleus, *FA* fatty acids, *LH* lateral hypothalamus, *NTS* nucleus tractus solitaries, *PVN* paraventricular nucleus, *SCN* supra chiasmatic nucleus, *VMN* ventromedial nucleus

Systemic energy balance, reflected in the maintenance of a stable body weight, is driven by an equilibrium between energy expenditure, i.e., energy consuming metabolic processes and energy supply which necessitates the existence of acute, short, and long-term energy sources and stores, respectively (Fig. 1). In mammals, the largest and most stable component of total energy expenditure (TEE) is the basal metabolic rate (BMR) which in humans makes up about 60–70% of total energy expenditure. Thermogenesis usually is defined as diet induced thermogenesis, typically around 10% of TEE in humans. Cold induced thermogenesis is an important contributor to TEE in small mammals. In mice it makes up around 30% of TEE at room temperature (around 20 °C) which is below their thermoneutral zone. In humans this is of minor importance due to appropriate clothing and indoor heating, which keep us within our thermoneutral zone. Activity or exercise-related energy expenditure is the most variable component of TEE. Humans are able to increase their energy expenditure over 20 fold during short term, vigorous bouts of exercise but over prolonged periods of days or weeks, activity energy expenditure rarely exceeds 50% of TEE and in normally active humans it is around 30% of TEE [18].

Long-term energy stores in mammals consist of lipids, stored in form of lipid droplets mainly in adipose tissue, whereas carbohydrates, such as circulating glucose and hepatic glycogen serve as rapidly available, short-term energy stores. The energy density of carbohydrates (around 4 kcal/g) is much lower than that of lipids (around 9 kcal/g), and carbohydrates are highly hydrated in contrast to lipids. As a result, the isoenergetic weight burden of carbohydrates is about 10 times higher than that of lipids which might explain why almost all animals rely on lipids as the major quantitative, long term storage form of energy [19]. Proteins, i.e., amino acids can also be used as energy substrates, especially during prolonged starvation when muscle protein is metabolized.

A long-term disequilibrium between energy intake and energy expenditure forcibly results in body weight changes. A positive energy balance with an energy intake exceeding energy expenditure leads to an increased body weight by accumulation of body fat, while a negative energy balance, e.g., during starvation or periods of increased physical activity leads to mobilization of energy stores and substrates resulting in a loss of body weight. An equilibrated energy balance relies on a highly complex and redundant central system of appetite regulation assuring energy supply via nutrition in form of the macronutrients. This central appetite regulation involves homeostatic and hedonic hypothalamic and corticolimbic neural circuitries that are controlled by interoceptive signals of nutrient availability as peripheral, afferent signals [20]. These internal signals comprise hormones such as leptin, ghrelin, insulin and gut hormones, as well as metabolites such as glucose and amino acids which all display daily oscillations. Their role in the crosstalk between energy metabolism and the circadian system has been extensively reviewed before [4, 7] and will not be discussed in detail here.

As depicted in Fig. 1, maintenance of energy balance involves both signals for long-term regulation of body weight or rather body fat mass, and short-term regulation of food intake in a diurnal manner. There is thus a coexistence of two different kind of processes: tonic ones that are enduring and relatively stable over days and episodic ones which vary in strength during the course of a day. The discovery of leptin in 1994, as a hormone secreted by adipose tissue, proved the existence of the long time postulated adipostatic regulation of body weight with leptin as a tonic signal of adipose tissue abundance mediating the inhibitory influence of fat on brain mechanisms. Episodic signals arise mainly as a consequence of food consumption and include both appetite stimulating and appetite inhibiting hormones and metabolites [21, 22].

## Metabolic rhythmicity and energy homeostasis

The mammalian circadian timekeeping system comprises a hierarchy of interconnected oscillators from the cellular to organismal level with a central clock located in the hypothalamic SCN which coordinates extra-SCN oscillators into a coherent timekeeping system [23]. The SCN as the master pacemaker is synchronized by photic cues (light) received directly from the retina as the main environmental zeitgeber. It thus acts as the hypothalamic link between the retina and oscillators in peripheral organs, entraining them to the 24 h light/dark cycle [24]. On a cellular level, the core molecular clock mechanism consists of an autoregulatory, cell autonomous system of transcriptional-translational negative feedback loops with the transcription factors CLOCK and BMAL1 at its center. It drives gene expression rhythms in thousands of genes that are involved in metabolism, immune function, cell proliferation, cancer and signaling. Almost all body cells express clock genes with the capacity to generate circadian oscillations [25].

In the strict sense of the definition circadian rhythms are only those whose endogenous, self-sustainable nature has been experimentally proven by showing that they are free running in the absence of external cues. Diurnal rhythms on the other hand are daily fluctuations that are synchronized with the day/night cycle, and are usually, but not necessarily, based on circadian rhythms that are entrained by an environmental zeitgeber. The term diurnal is somewhat ambiguous: it can refer to species that are active during day time (such as humans) in contrast to nocturnal species that are active during night time (such as mice and most rodent species). Here we use “diurnal” in the sense of daily metabolic and behavioral fluctuations that are synchronized with the day/night cycle.

The most obvious diurnal rhythm which governs daily systemic metabolic fluctuations is the sleep/wake cycle, because it determines daily fluctuations in energy intake (feeding/fasting) and energy expenditure (activity/resting) and defines the temporal ecological niche of an animal. The feeding/fasting rhythm in turn leads to metabolic fluctuations of hormones and metabolites which feed back on the circadian system by providing endogenous, peripheral zeitgebers that entrain peripheral oscillators in different organs such as liver, kidney and muscle, and endocrine organs. This can lead to a remodelling or even reprogramming of endocrine and metabolic signalling. It is important to emphasize that food intake rhythms are able to reprogram the clock of peripheral tissues and organs important for energy metabolism. Feeding time appears to be the dominant zeitgeber in these peripheral tissues, most importantly in the liver [5, 7, 9, 11]. Moreover, macro- and micronutrients have the ability to function as zeitgebers for the clock by activating or modulating specific clock proteins, affecting synchronicity between the central pacemaker and metabolically active peripheral tissues [26]. Metabolic processes, energy balance and the circadian time keeping system are thus highly interconnected (Fig. 1).

## Implications of the opposite diurnal rhythm in rodents and humans

It is important to be aware that most studies on diurnal and circadian aspects of metabolic regulation and energy balance are conducted in mice and rats. These are nocturnal species with their main activity and feeding period during the night. During daytime, in the time frame when human researchers are active and conducting most studies, these nocturnal species are actually in their resting period, that is in an overall catabolic state characterized by the mobilization of stored substrates such as glycogen and fat to fuel resting energy expenditure. Importantly, the molecular mechanisms and rhythmicity of the SCN clock operate in the same way in nocturnal and daytime active species. The daily rhythm of the hormone melatonin, which is tightly regulated by the SCN, also shows the same pattern in all mammals, i.e., high levels at night [4]. Melatonin is produced from serotonin by the pineal gland, a small neuroendocrine organ whose main function is the nighttime secretion of melatonin, a function which is highly conserved throughout the animal kingdom. Melatonin is considered as the chemical correlate of darkness conveying information about the duration of the night in both nocturnal and daytime active species. As an endocrine zeitgeber, melatonin regulates sleep/wake cycles and feeds back on the SCN for entrainment of circadian rhythms [27].

However, melatonin is an exception among the metabolic acting hormones that show diurnal oscillation. According to the sleep/wake cycle and resulting feeding/fasting cycles, most hormonal rhythms including that of glucocorticoids, leptin and ghrelin are in opposite phase in nocturnal and day time active species in relation to the day/night cycle. For example, both in rodents and humans the peak of circulating glucocorticoids is phase-locked with the onset of activity, that is at dusk and dawn, respectively [4, 9].

The SCN is also bidirectionally connected to other hypothalamic nuclei, such as the arcuate nucleus (ARC) and the ventromedial nucleus (VMN), areas that are important in appetite regulation (Fig. 1). These areas are also food entrainable and convey signals to peripheral organs [7]. For example, brown adipose tissue (BAT) thermogenic function was shown to be under the control of the VMN circadian clock which computes light and feeding inputs to modulate basal energy expenditure independently of the SCN [28].

## Feeding and exercise as important cues for diurnal entrainment

According to their nocturnal activity pattern, mice kept under a 12/12 h light/dark schedule show voluntary wheel running activity almost exclusively at night and also consume most of their food (about 75%) during the dark phase [29, 30]. The liver as the central metabolic organ and hub of nutrient distribution is particularly affected by the rhythmicity of food intake. Interestingly, it has been shown that manipulation of the feeding rhythm in mice did not affect oscillations of the hepatic core clock genes but changed the oscillating expression pattern of over 70% of hepatic genes including genes for key enzymes of glucose and lipid metabolism [29]. On the other hand, it is undisputed that hepatic physiology is highly controlled by its circadian clock. In different mouse models it was shown that mutations of core circadian clock components as well as forced changes in feeding time are closely associated with a range of metabolic disorders including the development of fatty liver and non-alcoholic fatty liver disease (NAFLD). Importantly, also in humans there is increasing evidence that circadian misalignment has adverse metabolic and cardiovascular consequences [31]. This seems fairly obvious, since beside the liver, all major metabolic organs including brown and white adipose tissue, pancreas, skeletal muscle, and intestine possess circadian clocks that regulate their specific physiological and metabolic functions [7, 9].

Skeletal muscle, as the major organ of energy expenditure, displays diurnal oscillations of multiple metabolic features affecting energy balance. For example, in lean, healthy humans, skeletal muscle mitochondrial respiration showed highest values late in the evening (11 pm) and lowest values at night (4 am) which reflected whole body energy expenditure [32]. Whole muscle lipid composition also showed variations across the day-night cycle [33]. The same group showed recently that in older, overweight, and metabolically compromised individuals the diurnal rhythmicity in skeletal muscle mitochondrial oxidative capacity and molecular clock gene expression was disturbed [34], again linking metabolic disorders with impaired diurnal rhythmicity. Of note, physical exercise, which is one of the most potent ways for improvement of muscle insulin sensitivity, is also a strong external zeitgeber for entrainment of the peripheral muscle clock. In mice it was shown that muscle contractions, as part of exercise, are sufficient to shift the muscle circadian clock phase [35]. Since nutrition is an important zeitgeber as well, it was recently suggested that there might be an optimal time for exercise in relation to meal timing for maximal improvement of muscle and whole body insulin sensitivity and glucose homeostasis [36]. Applying circadian principles to exercise interventions is thus considered to hold potential for improving exercise outcomes not only for patients, but also for healthy subjects and even elite athletes [37].

## Mitochondria and diurnal energy homeostasis

The temporal patterning of energy metabolism over the day implies diurnal fluctuations in mitochondrial activity and functionality, and it is well recognized that mitochondrial bioenergetics are controlled by the circadian clock as shown in a number of loss of function studies [7, 10, 11, 38]. Many metabolically relevant aspects of mitochondrial function, composition, and morphology show daily oscillations such as lipid composition, ATP production, respiration rates, production of reactive oxygen species (ROS), and mitochondrial fission–fusion cycles [7]. Almost 40% of liver mitochondrial proteins show oscillations and exhibit a diurnal pattern of accumulation, including key mitochondrial metabolic enzymes [39]. This likely involves transcriptional and post-transcriptional mechanisms as well as post-translational modifications [11]. For example, the circadian control of the bioavailability of nicotinamide adenine dinucleotide (NAD^+^, an important cofactor in oxidative metabolism) was shown to modulate liver mitochondrial oxidative function via protein acetylation, thus reflecting organismal metabolism across the daily cycles of fasting and feeding [40].

Regarding energy metabolism, there is now increasing interest in the crosstalk between different organs and tissues as well as between mitochondria and cellular functions which ultimately reflect back on organismal metabolism. Especially the role of mitokines as mediators of mitohormesis in the signaling between mitochondrial function and organismal physiology and health has been a recent focus of interest [12, 14, 41, 42]. However, the possible role of mitokines in circadian biology and their effects on diurnal energy balance have received little attention so far. Nevertheless, this role is certainly of considerable importance for understanding the metabolic function of mitokines as evident by the recent discovery of GDF15 as a regulator of diurnal energy balance [43] as discussed in detail later.

## Induction of mitokines by mitochondrial dysfunction

The importance of mitochondria in energy metabolism is underlined by the fact that mitochondrial dysfunction is linked to a number of severe diseases including myopathies, neurological, and cardiovascular disorders [7]. Cellular stress, including mitochondrial stress, activates the integrated stress response (ISR), a common adaptive pathway of eukaryotic cells for the restoration of cellular homeostasis in response to diverse stress stimuli which aims to optimize the cellular stress response. The ISR thus plays an important role in cell survival and cell death. Activation of the ISR converges on the phosphorylation of eukaryotic translation initiation factor 2 (eIF2α) which results in a reduction of overall translation while selectively favoring the translation of proteins implicated in stress recovery, such as ATF4 (activating transcriptional factor 4), the main regulator of the mitochondrial stress response in mammals [12]. Activation of ATF4 induces a general retrograde response that regulates cellular metabolism and mitochondrial function as a cellular adaptative stress response [44]. Among the genes known to be induced by activation of the ISR and ATF4 are the cytokines FGF21 and GDF15 [15]. Both are considered as mitokines induced in response to mitochondrial but also ER stress and other cellular stress situations in various organs and tissue; and both have been demonstrated to exert systemic metabolic effects [12, 14, 45]. FGF21 and GDF15 are used as biomarkers for the diagnosis and severity assessment of mitochondrial diseases including mitochondrial myopathy, [46, 47], but they were also shown to be elevated in patients with different inherited metabolic diseases that are not restricted to mitochondrial disorders [48]. Interestingly, circulating FGF21 and in particular GDF15 increase with aging, and both have been suggested as markers of biological age in humans [49].

## Mitokines as a double-edged swords in health and disease

Although they are markers of cellular stress and disease, FGF21 and GDF15 both have been shown to elicit metabolic improvements and even lead to increased longevity when over-expressed in mice [50, 51]. They are, therefore, thought to act as rescue or survival factors with a protective function aiming to restore metabolic homeostasis [14]. In a mouse model of adipocyte specific defect in oxidative phosphorylation (OXPHOS) both FGF21 and GDF15 were found to be induced and both were shown to be instrumental in protection from obesity and insulin resistance induced by high fat diet feeding [52]. On the other hand, these cytokines might also contribute to disease progression depending on the type and severity of disease. For example, the catabolic effect of GDF15 has been linked to cancer cachexia, and inhibition of GDF15 activity by targeting its receptor was shown to reverse cancer cachexia in mice [53]. In a recent review article the adaptive and maladaptive metabolic consequences of increased GDF15 expression and secretion in acute and chronic cellular stress situations have been discussed in detail [54]. Regarding FGF21, it seems to be an important agent in muscle atrophy. The precocious senescence and muscle loss of mice with muscle-specific deletion of the mitochondrial fusion protein OPA1 (optic atrophy protein 1) was rescued by muscle specific deletion of *Fgf21* [55]. Muscle loss due to prolonged fasting could also be prevented by muscle specific deletion of *Fgf21* in mice [56]. Of note, circulating FGF21 and GDF15 levels were elevated in COVID-19 patients hospitalized due to a severe acute respiratory syndrome coronavirus 2 (SARS-CoV-2) infection, and were found to be correlated with disease severity and mortality [57, 58].

## Metabolic role of FGF21

FGF21 is an endocrine acting member of the FGF subfamily that acts through the FGF receptor 1 (FGFR1) with the co-receptor β-Klotho (Klb) which determines the tissue specificity and metabolic action of FGF21 [59]. Its metabolic actions were first reported about 15 years ago, making it a therapeutic candidate in diabetes and obesity [60]. Since that time, the physiological and pharmacological effects of FGF21 and synthetic analogues have been intensively studied. FGF21 is considered as a hepatokine under normal physiological conditions, when liver is the main source for circulating levels. However, by various physiological stress conditions it can be induced in a variety of tissues, where it often acts in an auto/paracrine fashion. Hepatic expression and secretion of FGF21 is also induced by a broad variety of nutritional stress situations including ketogenic diets and starvation, amino acid restriction, simple sugars, and alcohol. It appears to play a protective role for the liver itself but also has endocrine effects such as increasing glucose uptake and adiponectin secretion from adipose tissue (for reviews see [61, 62]).

There is still an ongoing debate about the significance of central versus peripheral effects of FGF21. First evidence for a central action of FGF21 was obtained in a transgenic mouse model with hepatic overexpression of *Fgf21* leading to supraphysiological levels of circulating FGF21. Bookout et al. showed that actions in the SCN and dorsal vagal complex of the hindbrain mediated the starvation adaptation effects of FGF21 such as increased systemic glucocorticoid levels, altered circadian behavior, decreased body weight and insulin levels. Mice lacking the *Klb* gene in these brain regions were refractory to these effects of FGF21. The authors suggested that FGF21 does not affect central clock genes but possibly SCN output [63]. Later it was shown that FGF21 action in the brain also decreased sweet and alcohol preference and stimulated water intake in mice and rats suggesting a role of FGF21 in the maintenance of water balance in nutritional stress situations that could lead to dehydration [61]. It should be noted, however, that most of the studies on central effects of FGF21 were conducted using *Fgf21* transgenic mice or pharmacological administration of FGF21, that is in situations of supraphysiological FGF21 concentrations. There are also differences between the physiological effects of endogenous FGF21 and pharmacological action of recombinant FGF21 which might be due to complex dose response relationships [62].

## Diurnal oscillations of FGF21

*Fgf21* expression in mice shows a diurnal rhythm and the gene is thought to be directly controlled by first order clock-controlled transcription factors [64]. It was first shown that treatment of mice with an agonist of peroxisome proliferator-activated receptor α (PPARα, a transcription factor known to induce *Fgf21* expression) induced diurnal fluctuations in hepatic *Fgf21* expression [65]. Circadian oscillations of hepatic *Fgf21* expression were later confirmed and it was found that the transcriptional repressor E4-binding Protein 4 (E4BP4, a clock-controlled transcription factor) regulated hepatic *Fgf21* during circadian cycles and feeding in mice. E4BP4 mediated the suppression of *Fgf21* expression by feeding which could be mimicked by insulin treatment [66]. It should be noted, however, that in these studies plasma FGF21 levels were not analyzed. Furthermore, hepatic FGF21 expression and secretion in mice is affected by nutrition-related cues such as fasting, free fatty acids as well as low protein diets or amino acid restriction, which all induce its hepatic gene expression [64] and might interfere with the circadian regulation.

In humans, daily fluctuations in circulating FGF21 are well documented. It was reported that the 24-h oscillatory pattern of circulating FGF21 resembled that of free fatty acids and cortisol with a peak in the early morning and lowest levels in the afternoon, and was opposite to the patterns of insulin and glucose [67]. In healthy females subjected to a 72-h fasting, circulating FGF21 followed a circadian rhythm independent of glucose or free fatty acid levels and the authors concluded that the circadian regulation apparently has a stronger impact on plasma FGF21 than the fasting status [68]. This is in line with findings that, in contrast to mice, hepatic FGF21 expression is not induced by short term fasting but only after prolonged starvation in humans [69].

## GDF15 as a stress-induced cytokine with metabolic action

First identified in 1997, GDF15 was labelled as a divergent member of the transforming growth factor β (TGFβ) superfamily [70, 71]. Extensive literature describes GDF15 as a biomarker for a number of diverse pathologies or conditions such as diabetes, cardiovascular disease [72], obesity [73, 74], cancer [75, 76], mitochondrial disease [77], and aging [78]. For example, in old hospitalized patients elevated GDF15 concentrations are associated with lower measures of muscle mass and strength in men [79]. In the very old GDF15 was found to be a predictor of mortality showing an especially strong increase in centenarians [49]. Therefore, also considering that under normal circumstances *Gdf15* shows low expression levels in most organs, GDF15 is regarded mainly as a stress-induced hormone/protein. However, the functional consequences in humans are not yet understood. Recently, it was suggested that GDF15 provides some protection from aging-mediated systemic inflammation. On one hand, GDF15 was found to correlate positively with aging associated systemic inflammation in humans. On the other hand, aged *Gdf15* ablated mice showed increased markers of hepatic and adipose tissue inflammation as well as a slightly deteriorated glucose homeostasis [80]. This could possibly be linked to GDF15 effects on macrophages. Using different mouse models, it was shown that a reduced oxidative capacity of macrophages results in systemic insulin resistance and adipose inflammation. This could be reversed by treatment with GDF15 which improved oxidative function of macrophages leading to their M2-like polarization [81].

In recent years, the metabolic role of GDF15 has received increasing attention. So far, the control of food intake, and, therefore, of energy metabolism is regarded as its most important metabolic role. Mice that are over-expressing or treated with recombinant GDF15 are resistant to the development of diet induced obesity and associated metabolic disorders (see [12] for review). However, *Gdf15* ablated mice show only slightly increased body weight and adiposity and minor alterations in energy metabolism suggesting that it does not play a major role as a physiological regulator of appetite and body weight in healthy animals [43, 82, 83]. Cellular targets of GDF15 and its action at the molecular level are still under debate, partly because a specific receptor for GDF15 was identified only recently [84].

## GFRAL as the unique receptor for GDF15 signaling

In 2017, GDNF receptor alpha-like (GFRAL), an orphan receptor of the glial-derived neurotrophic factor (GDNF) receptor α family, was described as the unique receptor for GDF15 signaling that was necessary for reduction of food intake and body weight after pharmacological application of GDF15 by signaling through the tyrosine kinase co-receptor Ret. While Ret is widely expressed in multiple cells and tissues, GFRAL expression, at least in mice, was found to be specific to the area postrema (AP) and nucleus of the solitary tract, (NTS) in the hind brain ([85–88]. In humans a substantial expression of GFRAL mRNA was recently demonstrated in adipose tissue, predominantly occurring in preadipocytes. The authors also reported a modest but statistically significant increase of lipolysis in human adipose tissue explants upon treatment with recombinant human GDF15 which was abrogated by addition of a neutralizing GDF15 antibody [89]. This suggests that the expression pattern of GFRAL might be different in mice and humans.

The precise mechanisms and pathways for regulation of energy balance by GDF15 are only starting to emerge. Importantly, the hindbrain AP/NTS is a brain region of special interest regarding brain integration and processing of peripheral signals because of its location outside the blood–brain barrier. The AP is a circumventricular organ, receiving a large amount of signals from the periphery due to a high vascularization in this region [90]. The AP/NTS area is also part of the dorsal vagal complex, an important component of the gut-brain signaling axis and expresses receptors of a number of hormones involved in satiety and food intake regulation such as amylin [91, 92] or adiponectin [93]. One of the well described actions of the AP is the control of nausea and vomiting [94], but it has also been long known as a control center of food intake [95]. This is in line with mounting evidence that GDF15 could cause food aversion and malaise as an endocrine signal of nutritional stress [96] and the recent finding that central delivery of GDF15 into rats and shrews induced behaviors indicative of nausea and emesis preceding the onset of anorexia [97].

The nature of the GFRAL-positive neurons in the AP/NTS is not yet clear. Recently it was shown that GFRAL is located in a subset of neurons containing the neuropeptide cholecystokinin (CCK) which are spanning the AP/NTS region. Targeted deletion of these CCK neurons as well as pre-administration of a CCK-receptor antagonist abrogated the anorectic effect of GDF15. The authors suggested that the primary target for GDF15 is a distinct population of GFRAL/CCK neurons which span the AP/NTS to engage the neural circuitry involved in anorexia and conditioned taste aversion [98]. Nevertheless, the physiological mechanisms by which GDF15 signaling via GFRAL modulates food intake as well as its possible interaction with other appetite regulating hormones and brain regions are yet unclear.

## GDF15 in appetite and body weight regulation

Under basal conditions GDF15 does not seem to play a role in food preference. It was recently reported that neither *Gdf15* nor *Gfral* ablated mice showed significant alterations in their relative food choice when presented with a choice of pure macronutrients. On the other hand, pharmacological GDF15 administration selectively reduced the preference for fat in wildtype mice [99] and injection of GDF15 lead to a preference for plain water against water supplemented with saccharin [96]. Importantly, similar to studies with FGF21, the strong anorectic effect of GDF15 as well as its induction of nausea and food aversion were observed only after pharmacological treatment with GDF15. Furthermore, the body weight lowering effect of GDF15 is apparently not due to its anorectic effect alone. GDF15 has been linked to cancer cachexia and a recent study found that inhibition of the GDF15-GFRAL activity by a GFRAL targeting antibody reversed cancer cachexia in mice [53]. Mechanistic exploration suggested that GDF15 induced a lipolytic response in adipose tissue, independently of anorexia, which was mediated by the peripheral sympathetic nervous system (SNS). The authors showed that peripheral chemical sympathectomy as well as prevention of lipolysis by knockout of adipose triglyceride lipase (ATGL, gene name *Pnpla2*) protected mice from GDF15-induced weight loss [53].

## Circadian control of GDF15

Similar to *Fgf21*, *Gdf*15 also seems to be an oscillating, clock controlled gene as first shown by studies on rat uterus endometrial stromal cells (UESCs) [100]. Subsequently, it was shown that the *Gdf*15 mRNA expression in UESCs was enhanced after treatment with an antagonist of REV-ERBα, a transcription factor of the core clock machinery, and that REV-ERBα represses *Gdf*15 expression by direct binding to circadian clock-controlled cis-regulatory elements in its promoter region [101]. More recently, a RNAseq analysis of skin fibroblasts derived from individuals with type 2 diabetes identified *Gdf15* among over 1000 genes that were differentially expressed according to the individual chronotype, which was different between diabetics and non-diabetics [102]. Interestingly, *Gdf15* is highly expressed in the pineal gland, where it shows clear day/night differences. In a microarray analysis of rat pineal glands collected either at midday or midnight *Gdf15* mRNA expression was found to be over 12 times higher at night compared to day and thus among the top 40 genes with the highest day/night expression differences [103]. So far, there are only limited data on diurnal variations of circulating GDF15 levels in humans. Data from an Asian cohort consisting of 14 healthy individuals undergoing periodical measurements over a single 24-h cycle suggest that circulating GDF15 levels vary in a diurnal pattern [104]. In 12 out of the 14 individuals GDF15 levels showed a peak around midnight and a nadir (minimum levels) approximately at noon.

## FGF21 and GDF15 as myomitokines

The first suggestion of FGF21 as a mitokine dates from 2013 when Kim et al. showed that skeletal muscle–specific deletion of Atg7 (autophagy-related 7) induced autophagy deficiency in mice led to the protection from obesity and insulin resistance by inducing Fgf21 [105]. Since then, in different mouse models of skeletal muscle mitochondrial dysfunction the role of FGF21 and GDF15 as mediators of mitohormesis has been extensively documented leading to their labeling as myomitokines [12, 106].

Importantly, disturbance of either mitochondrial fission or fusion in skeletal muscle was found to lead to muscle atrophy and induction of FGF21 [55, 107, 108], which was alleviated by the simultaneous inhibition of fusion and fission processes [109]. As already mentioned, there is mounting evidence that muscle FGF21 is directly involved in the regulation of muscle mass and function by affecting the anabolic/catabolic balance possibly in an auto/paracrine manner through induction of mitophagy [55, 56]. Mouse models, where an induction of both FGF21 and GDF15 was demonstrated include the Deletor mouse, a model for progressive mitochondrial myopathy [15], mCrif1 (CR6-interacting factor 1)-Ko mice with skeletal muscle specific disruption of oxidative phosphorylation [110], Aifm1 (apoptosis-inducing factor mitochondrion-associated 1)-knock-in mice, a model of severe myopathy [111], and, as the best studied muscle mitohormesis model, UCP1-tg mice [112, 113]. The latter is a transgenic mouse model with skeletal muscle directed, low expression levels of the mitochondrial uncoupling protein 1 (UCP1), which is usually only expressed in brown adipocytes [114]. This leads to a slightly compromised skeletal muscle mitochondrial function due to increased uncoupling of the respiratory chain [115]. Of note, in two independently generated mouse models it has been demonstrated that muscle-targeted respiratory uncoupling increases longevity and promotes healthy aging [17, 116]. Despite a decreased muscle mass and strength, UCP1-tg mice display a healthy metabolic phenotype characterized by increased energy expenditure, delayed diet-induced obesity development, reduced hepatic steatosis, browning of white adipose tissue, and improved glucose homeostasis, leading to an increased lifespan on obesogenic diets, and increased overall longevity [16, 17, 117, 118].

## Importance of FGF21 and GDF15 for the metabolic phenotype of UCP1-tg mice

A transcriptome analysis revealed *Fgf21* and *Gdf15* among the top upregulated genes in SM of UCP1-tg mice which translated into an over fivefold increase in their circulating levels [43, 112, 113]. Crossing of UCP1-tg mice with *Fgf21* and *Gdf15* ablated mice, respectively, showed that in this model FGF21 seems to be of minor importance for the beneficial metabolic phenotype compared to GDF15 [43, 119]. Loss of FGF21 prevented the browning, i.e., induction of thermogenic adipocytes in inguinal and visceral white adipose tissue (WAT) and also had a minor effect on circulating triglycerides, but it did not prevent obesity resistance and protection from developing a fatty liver, nor the improved insulin sensitivity observed in UCP1-tg mice [119]. On the other hand, *Gdf15* ablation revealed its major impact on the metabolic phenotype of UCP1-tg mice [43]. Of note, there were no apparent auto/paracrine effects of either FGF21 or GDF15 on skeletal muscle morphology, function or mitochondrial energetics suggesting their purely endocrine action when induced as myomitokines. In the UCP1-tg mouse model loss of GDF15 led to a progressive body mass increase which was exclusively due to an accumulation of body fat, while lean body mass was not affected. Further analyses relating to the metabolic remodeling phenotype of UCP1-tg mice showed that their WAT remodeling (browning) as well as increased insulin sensitivity were abolished upon loss of GDF15, interestingly despite their increased levels of circulating FGF21 which were not affected by loss of GDF15 [43].

## GDF15 and the diurnal regulation of energy balance

UCP1-tg mice are smaller and leaner than wildtype and, not surprisingly, show an increased weight specific energy expenditure. However, considering the assumed anorectic action of GDF15 it is rather puzzling that they also show an increased weight specific energy intake compared to wildtype [16] despite highly elevated circulating GDF15. Therefore, we conducted a comprehensive in vivo metabolic phenotyping with a high temporal resolution using indirect calorimetry combined with analysis of physical activity and feeding/drinking [43]. Interestingly, we observed a shift in the daily rhythm of food intake and energy expenditure in UCP1-tg mice which was prevented by ablation of *Gdf15*. Compared to wildtype, UCP1-tg mice showed a daytime-restricted suppression of food intake in line with a reduction of total energy expenditure during the day only, but an increased night time energy intake and expenditure. Ablation of *Gdf15* in UCP-tg mice prevented their day time restricted anorexia without affecting the physical activity pattern. When calculating overall energy balance, this loss of GDF15-dependent day time anorexia together with a preserved elevated night time energy intake proved sufficient to explain the progressive fat accumulation that we observed in *Gdf15* ablated UCP1-tg mice. As a consequence of the diurnal variation in energy balance, skeletal muscle mitochondrial stress in UCP1-tg mice apparently promotes a systemic metabolic flexibility as evident by an increased amplitude of the respiratory quotient (RQ), an indicator of relative substrate oxidation. This was also prevented by the ablation of *Gdf15*. Taken together, this was the first demonstration of a GDF15-dependent, diurnal anorexic response that reprograms systemic energy homeostasis and metabolic flexibility, of note in a model of endogenously elevated GDF15 [43]. Subsequent analysis of diurnal differences in the skeletal muscle stress response and GDF15 induction showed that, despite a similar activation of the ISR during daytime and at night, GDF15 gene expression and circulating levels were significantly higher at daytime (during the resting phase) than at night. Of note, circulating GDF15 concentrations at nighttime were consistently below 400 pg/ml, while daytime values were above this threshold.

**Fig. 2:**
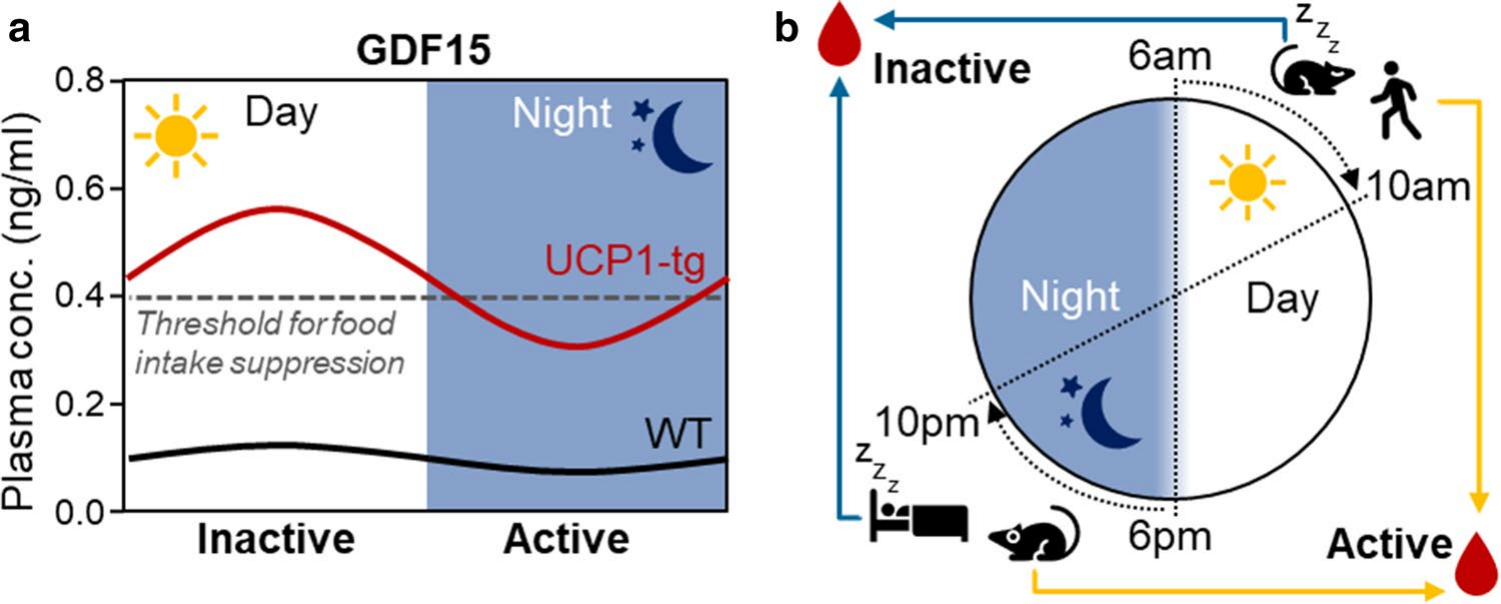
Diurnal variations of circulating GDF15 and future directions for translation from mice to man. **a** Hypothetical model of GDF15 action during mitochondrial stress. In wildtype mice circulating GDF15 shows daily oscillations with a low amplitude and levels that are below the threshold to induce central anorectic action. In the UCP1-tg mouse model of skeletal muscle mitochondrial uncoupling GDF15 is released from muscle with a magnified amplitude of the daily oscillation leading to daytime levels that exceed the threshold for food intake suppression. This results in a daytime restricted anorexia without affecting night time food intake. **b** Mice and humans show opposite diurnal activity rhythms resulting in phase shifted diurnal metabolic oscillations. Therefore, we suggest that in metabolic experiments blood and tissue samples from rodent models are best obtained in the evening after lights off to correspond to the early daytime activity phase of humans

Based on these data we propose that activation of *Gdf15* by mitochondrial stress amplifies its daily oscillations as a clock regulated gene, resulting in endogenous levels of GDF15 that exceed a certain threshold (here 400 pg/ml) only during the day, resulting in a diurnal, daytime restricted anorectic response, which apparently controls the systemic metabolic adaptations of this mouse model [43] (Fig. 2a). So far, we can only speculate on the mechanisms driving the increased diurnal rhythmicity of GDF15 expression as a stress-induced mitokine. An obvious origin could be the circadian nature of several aspects of mitochondrial function as already discussed. For example, fluctuations in ROS levels resulting from fluctuations in mitochondrial respiration rates might have retrograde effects on gene expression levels, but this still warrants investigation.

Interestingly, in the aforementioned human study on diurnal variations of GDF15, calculated peak values at night (i.e., during sleep/resting period) were around 420 pg/mL and minimum daytime values around 340 pg/ml [104]. This is strikingly similar to the described mouse data considering the reversed diurnal rhythm in mice compared to humans.

## Conclusions

Metabolic fluxes and demands that ultimately determine energy balance show pronounced temporal rhythmicity in alignment with the 24 h diurnal rhythm of feeding and activity periods versus fasting and resting periods. The intrinsic circadian timekeeping system serves to control and optimize the temporal fluctuations of metabolism and it is well known that circadian misalignment imposed by modern lifestyle factors, such as jet lag, night and rotating shift work, is a major contributor to global health problems including obesity related pathologies [31]. Conversely, time-restricted feeding, i.e., limiting food intake to a period of 12 h maximum daily during the activity phase, has been suggested as a promising tool for circadian and metabolic improvements in humans [120].

**Fig. 3:**
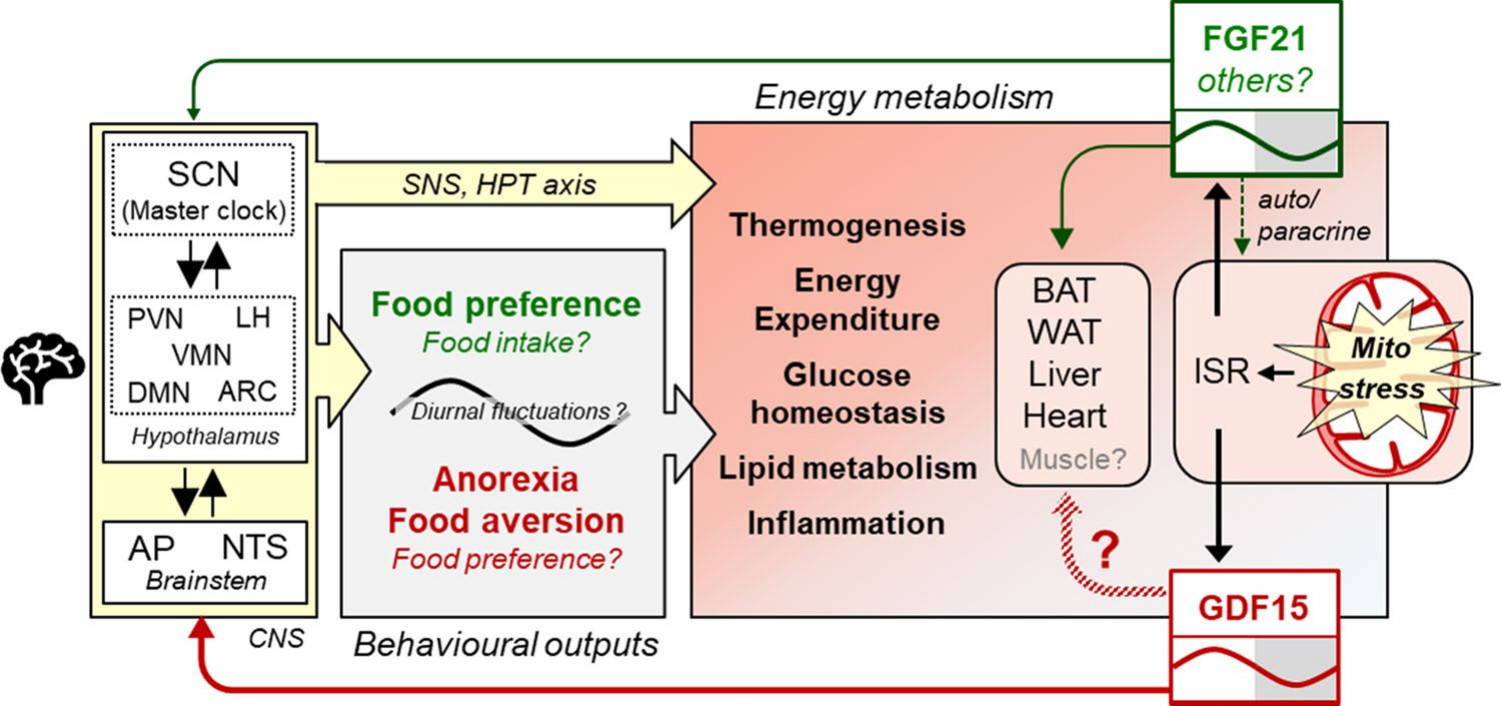
GDF15 and FGF21 as mitokines in the diurnal regulation of energy balance. Mitochondrial dysfunction induces GDF15 and FGF21 that act as endocrine mediators of mitohormesis by affecting central appetite regulation and energy balance. There is evidence that FGF21 acts directly in the brain and modulates SCN output, although the exact neuronal targets and pathway are not yet known. Central actions of FGF21 include behavioral changes such as an altered food preference as well as effects on thermogenesis and energy expenditure possibly through an increased SNS output and activation of the HPT axis. FGF21 also has direct effects on BAT, WAT, liver and heart. For GDF15 effects on energy and substrate metabolism are thought to be mainly centrally regulated, possibly also by increasing SNS output. GFRAL, the only known specific GDF15 receptor was only identified in neurons of the AP and NTS region in the hindbrain. Supraphysiological levels of GDF15 induce anorexia, probably induced by a visceral malaise state leading to food aversion. Direct peripheral effects of GDF15 are also discussed but still controversial. In mice, circulating levels of FGF21 and GDF15 as mitokines both show similar diurnal fluctuations with increased levels in the resting phase (daytime) resulting in a GDF15 driven, daytime restricted anorexia. *BAT* brown adipose tissue, *GFRAL* glial-derived neurotrophic factor receptor alpha-like, *HPT* hypothalamus–pituitary–adrenal axis, *ISR* integrated stress response, *SNS* sympathetic nervous system, *WAT* white adipose tissue, for other abbreviations see Fig. 1

The beneficial effect of time-restricted feeding has been demonstrated numerous times in human trials and animal studies [121, 122]. In animal studies this is achieved simply by restricting food access to a certain time-window. Intriguingly, the day time restricted GDF15 induced anorexia leads to a “voluntary” time restricted feeding in the UCP1-tg mouse model. This demonstrates a clear link between mitokine induction, circadian physiology and metabolic health. Figure 3 summarizes our current knowledge about the role of FGF21 and GDF15 as mitokines in the regulation of energy regulation. Interestingly, FGF21 and GDF15 both show diurnal fluctuations and directly target brain regions involved in appetite and circadian regulation. However, the metabolic significance of their rhythmicity has received little attention so far.

Surprisingly, although diurnal fluctuations in circulating FGF21 have been known for some time, it is not yet known if and how these fluctuations are affecting temporal patterns of feeding behavior, food preference or substrate metabolism. Several years ago, it was shown that overexpression of FGF21 leading to continuously elevated circulating levels in transgenic mice altered their wheel-running behavior by suppressing the output of the SCN, which further resulted in lower insulin levels and growth inhibition [63]. However, the metabolic implications and the role in circadian biology of physiological oscillations of FGF21 as a mitokine have not yet been addressed to the best of our knowledge.

Furthermore, possible interactions of GDF15 and FGF21 are likely, but have not been considered yet. Protein restriction, which increases energy expenditure but also food intake in mice, potently induces hepatic FGF21 expression and secretion. The metabolic effects of protein restriction are absent in FGF21-deficient animals, suggesting that FGF21 is responsible for these effects [123]. This implies an appetite inducing, orexigenic effect of FGF21, in contrast to the well described anorectic action of GDF15. If these effects are possibly counteracting each other in models, where both FGF21 and GDF15 are elevated or if there are additional mitokines involved is still completely unknown.

## Future perspectives

The metabolic significance of diurnal mitokine oscillation in the regulation of energy balance is just starting to emerge. We need more data on diurnal variations of mitokines and the underlying molecular mechanisms from different models of mitochondrial disturbances ranging from mitohormesis models such as the UCP1-tg mouse to disease models such as the Deletor mouse. Mitokine target tissues, inter-tissue communication and time-dependent action of mitokines also remain elusive and require further investigations, as well as the impact of diet and its macronutrient composition on diurnal patterns of mitokines.

On a general note, regarding the relevance of diurnal fluctuations of metabolic hormones and processes for the regulation of overall energy balance, this aspect deserves better attention in the investigation of mouse models. In animal studies biological samples are usually obtained at one time point only, mostly in the morning, that is during the transition from active to resting state. To optimize translation from mice to men, such samples should ideally be obtained (additionally) at the beginning of the activity period, that is in the evening shortly before or after lights off (see also Fig. 2b). Also, the increasing use of automated in vivo metabolic phenotyping systems that monitor activity, feeding/drinking, energy expenditure, etc. with a high temporal resolution should provide excellent opportunities to include the important aspect of circadian/diurnal biology in animal studies on energy balance, obesity, and metabolic disorders.
